# Quantifying functional connectivity: The role of breeding habitat, abundance, and landscape features on range‐wide gene flow in sage‐grouse

**DOI:** 10.1111/eva.12627

**Published:** 2018-05-12

**Authors:** Jeffrey R. Row, Kevin E. Doherty, Todd B. Cross, Michael K. Schwartz, Sara J. Oyler‐McCance, Dave E. Naugle, Steven T. Knick, Bradley C. Fedy

**Affiliations:** ^1^ School of Environment, Resources and Sustainability University of Waterloo Waterloo ON Canada; ^2^ U.S. Fish and Wildlife Service Lakewood CO USA; ^3^ Rocky Mountain Research Station USDA Forest Service National Genomics Center for Wildlife and Fish Conservation Missoula MT USA; ^4^ College of Forestry and Conservation University of Montana Missoula MT USA; ^5^ Fort Collins Science Center U.S. Geological Survey Fort Collins CO USA; ^6^ Forest and Rangeland Ecosystem Science Center U.S. Geological Survey Boise ID USA; ^7^ Present address: 2140 White Pine Pl. Boise ID 83706 USA

**Keywords:** dispersal, gene flow, genetic differentiation, genetic diversity, habitat selection models, isolation by resistance, landscape resistance

## Abstract

Functional connectivity, quantified using landscape genetics, can inform conservation through the identification of factors linking genetic structure to landscape mechanisms. We used breeding habitat metrics, landscape attributes, and indices of grouse abundance, to compare fit between structural connectivity and genetic differentiation within five long‐established Sage‐Grouse Management Zones (MZ) I‐V using microsatellite genotypes from 6,844 greater sage‐grouse (*Centrocercus urophasianus*) collected across their 10.7 million‐km^2^ range. We estimated structural connectivity using a circuit theory‐based approach where we built resistance surfaces using thresholds dividing the landscape into “habitat” and “nonhabitat” and nodes were clusters of sage‐grouse leks (where feather samples were collected using noninvasive techniques). As hypothesized, MZ‐specific habitat metrics were the best predictors of differentiation. To our surprise, inclusion of grouse abundance‐corrected indices did not greatly improve model fit in most MZs. Functional connectivity of breeding habitat was reduced when probability of lek occurrence dropped below 0.25 (MZs I, IV) and 0.5 (II), thresholds lower than those previously identified as required for the formation of breeding leks, which suggests that individuals are willing to travel through undesirable habitat. The individual MZ landscape results suggested terrain roughness and steepness shaped functional connectivity across all MZs. Across respective MZs, sagebrush availability (<10%–30%; II, IV, V), tree canopy cover (>10%; I, II, IV), and cultivation (>25%; I, II, IV, V) each reduced movement beyond their respective thresholds. Model validations confirmed variation in predictive ability across MZs with top resistance surfaces better predicting gene flow than geographic distance alone, especially in cases of low and high differentiation among lek groups. The resultant resistance maps we produced spatially depict the strength and redundancy of range‐wide gene flow and can help direct conservation actions to maintain and restore functional connectivity for sage‐grouse.

## 1 INTRODUCTION

Functional connectivity describes how landscapes influence the movement of individuals between habitat patches (Tischendorf & Fahrig, 2000). Its conservation is fundamental to the protection of diverse and viable populations able to persist and adapt to changing conditions (Fahrig & Merriam, 1985; Lamy et al., 2013; Morrissey & de Kerckhove, 2009). The distribution of habitat on the landscape (i.e., structural connectivity) often does not correlate directly with functional connectivity, because it does not consider the behavioral responses of individuals to habitat structure, nor their willingness to disperse through undesirable habitats. Landscape genetic approaches compare gene flow (i.e., functional connectivity) to connectivity measures and yield direct insights into the relative importance of landscape features and their configuration (Manel, Schwartz, Luikart, & Taberlet, 2003; Row et al., 2015).

Landscape features influencing genetic structure can range from natural barriers, such as mountains or lakes, to more recent barriers such as roads and development. Thus, insight from landscape genetics can help to guide management and conservation efforts by identifying specific features that reduce or facilitate gene flow and by identifying locations where mitigation of impedance is required (Epps et al., 2005; Roever, van Aarde, & Leggett, 2013). Despite the potential implications, the output from functional connectivity analyses is often omitted from conservation plans because they are not carried out in ways that are conducive to management objectives (Keller, Holderegger, van Strien, & Bolliger, 2015). For example, both the spatial extent of the study area and the resolution of landscape variables can influence the estimated importance of landscape features to functional connectivity (Anderson et al., 2010), which makes extrapolation across scales and comparison among studies challenging. Thus, conducting research at scales too large or too small for planning tools will hinder implementation (Keller et al., 2015). Furthermore, investigating both landscape variables that can be actively managed and those that are likely to impact connectivity, but cannot be actively managed (e.g., elevation), will likely lead to biologically meaningful results that can also be incorporated into conservation plans.

Although functional connectivity is likely influenced by a multitude of landscape features, it can prove challenging to disentangle their relative effects (Row, Knick, Oyler‐McCance, Lougheed, & Fedy, 2017). One way of modeling the biological importance of multiple landscape features is through the use of habitat indices. Habitat indices are typically derived from occurrence or radiotelemetry data and are used to predict the probability of occupancy across a landscape based on the suitability of habitat (Fedy et al., 2012; Phillips, Anderson, & Schapire, 2006) or the probability of selection for habitat types (Gubili et al., 2017; Shafer et al., 2012). Using habitat indices in landscape genetic models can assist with establishing a direct link between model results and management actions, and can alleviate some of the issues related to testing a multitude of landscape variables (i.e., type I error). If structural connectivity quantified using habitat indices proves to be a strong predictor of functional connectivity (Row, Blouin‐Demers, & Lougheed, 2010; Row et al., 2015; Wang, Yang, Bridgman, & Lin, 2008), identifying regions of importance to maintaining connectivity and predicting impacts from changes to habitat suitability become more clear (Roever et al., 2013). However, in some cases, the relationship between habitat indices and functional connectivity can be weak (Roffler et al., 2016). This lack of relationship is likely the result of a mismatch between dispersal and the habitats modeled in the indices (Ribe, Morganti, Hulse, & Shull, 1998; Spear, Balkenhol, Fortin, McRae, & Scribner, 2010).

Habitat models used in landscape genetic analysis generally do not include, nor model, variation in local population abundance. However, local population abundance can influence dispersal (Matthysen, 2005; Pflüger & Balkenhol, 2014; Strevens & Bonsall, 2011) and regional differences in population abundance can impact genetic drift and spatial genetic structure (Row, Wilson, & Murray, 2016). Therefore, the inclusion of effective population sizes in landscape genetic models can improve the fit between genetic differentiation and landscape variables hypothesized to affect genetic differentiation (Weckworth et al., 2013). For example, even when the probability of occupancy is high, low abundance could lead to increased genetic differentiation. Therefore, including abundance estimates in habitat indices should improve the prediction of functional connectivity. However, due to the difficulty in obtaining abundance information at large spatial scales, abundance is rarely incorporated into landscape genetic models.

In this study, we establish the drivers of functional connectivity for the greater sage‐grouse (*Centrocercus urophasianus*; hereafter sage‐grouse) across the species’ range in North America. Sage‐grouse are distributed across eleven US states and two Canadian provinces in western North America. However, there has been a 44% range contraction since European settlement and substantial declines in population size since the 1960s (Garton & Connelly, 2011; Schroeder et al., 2004). These decreases in range and population size have largely been attributed to habitat loss and fragmentation and have resulted in the species being petitioned for listing under the U.S. Endangered Species Act multiple times. Although populations are declining, there is a wealth of information available on the species’ habitat utilization (Doherty, Naugle, & Walker, 2010; Doherty, Naugle, Walker, & Graham, 2008; Fedy et al., 2014) and range‐wide estimates of abundance through counts of males at breeding leks (Doherty, Evans, Coates, Juliusson, & Fedy, 2016; Garton et al., 2011) where they congregate to attract and compete for access to females. Combining this habitat utilization and abundance information with our range‐wide genetic data makes sage‐grouse an ideal candidate for exploration of the link between habitat, abundance, and functional connectivity.

The relationship between functional connectivity and landscape or habitat variables is often quantified using regression‐based approaches in which pairwise genetic differentiation between groups or individuals is compared to pairwise landscape‐based resistance (or landscape cost) distances derived from a variety of single or combined landscape features (Cushman & Landguth, 2010; McRae, 2006; Van Strien, Keller, & Holderegger, 2012). Here, we used this approach to address three main questions. 1) Do habitat indices provide a better fit with genetic data than individual or combined landscape predictors? 2) Does the incorporation of abundance estimates improve the fit between genetic and habitat‐based resistance distances? 3) How well do the best fitting resistance models predict genetic differentiation across landscapes when compared to distance alone? Because the abundance and distribution of very low‐quality habitat can be disproportionately important for functional connectivity (Row et al., 2010, 2015), identifying these thresholds (Keller et al., 2015; Méndez, Vögeli, Tella, & Godoy, 2014) where functional connectivity is reduced could help focus conservation efforts on improving the habitat quality of landscapes to reach the necessary thresholds. Thus, we also determined whether there were consistent thresholds in habitat or landscape predictors which, when exceeded, resulted in disruption of functional connectivity for sage‐grouse. Overall, our results should provide guidelines for large‐scale evaluations of functional connectivity and assist with management goalms of maintaining or restoring functional connectivity.

## 2 MATERIALS AND METHODS

### 2.1 Study area and sample collection

**Figure 1:**
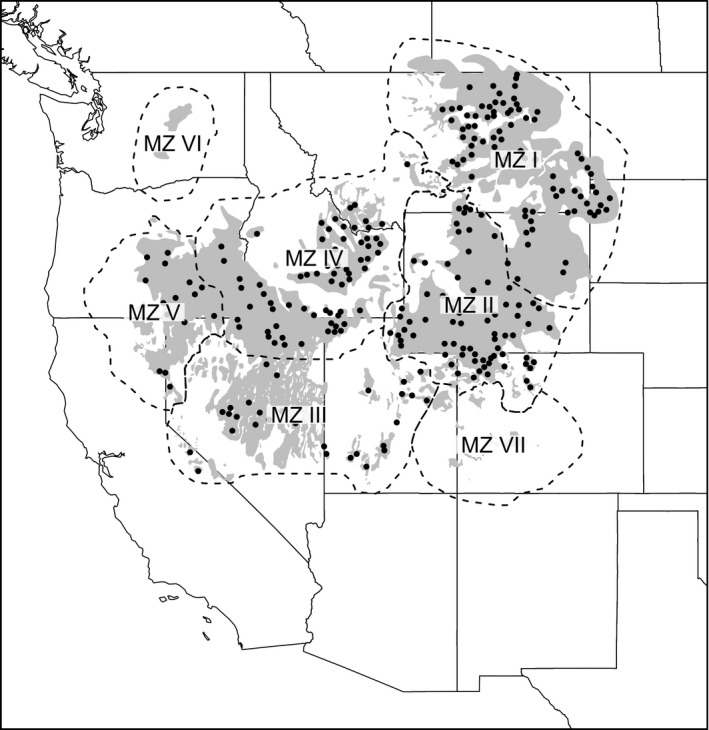
Locations (black circles) of 267 breeding lek clusters of greater sage‐grouse samples (6,844 samples in total) collected from 2005 to 2014 and used to compare genetic differentiation and landscape resistance. Full distribution of all samples can be found in Figure S1. Shaded gray area represents the current distribution of greater sage‐grouse, and dotted lines represent the extent of seven management zones for the species. Samples from management zones I–V were included in this analysis

Our study area includes nearly the entire range of sage‐grouse in North America, with the exception of a few small, geographically disjunct portions of the range which include individuals within the Canadian provinces of Alberta and Saskatchewan and sage‐grouse MZs VI and VII (Figure 1, Figure S1). The sage‐grouse range largely coincides with the distribution of sagebrush (*Artemisia spp*.) in western North America, an ecosystem that has become increasingly fragmented due to habitat conversion for agriculture, housing development, oil and gas development, wildfire, exotic grasses and invasive conifer encroachment (Knick et al., 2003). Within the range, we stratified our analysis based on long‐standing Sage‐Grouse Management Zones (here after MZ; Figure 1), which were established using differences in environmental attributes that influenced vegetation communities in each zone and were uninfluenced by administrative or governmental boundaries (Stiver et al., 2006). This stratification ensured relevance to previous studies, ongoing management initiatives, and consistency with the development of existing habitat and abundance layers (Doherty et al., 2016).

We used 6,844 spatially referenced feather and tissue samples collected across the sage‐grouse range (Figure 1, Table 1). Feather samples were collected noninvasively (Bush, Vinsky, Aldridge, & Paszkowski, 2005; Row et al., 2015) from leks, and blood samples were collected from captured sage‐grouse during the breeding season as part of radiotelemetry research efforts. Samples were collected from 1,392 leks from 2005 to 2014 by field staff from a variety of state and federal management agencies and nongovernmental organizations.

**Table 1:** Number of Greater Sage‐Grouse samples, breeding leks and grouped lek clusters used to establish patterns of genetic diversity and population structure in each of five long‐established management zones

Zone	Number of individuals included	Number of leks	Mean (*SD*) number samples per lek	Number of lek clusters	Mean (*SD*) individuals per cluster	Mean $G_{\mathrm{ST}}^{\prime }$ (*SD*)	Max $G_{\mathrm{ST}}^{\prime }$
MZ I	2,095	419	4.77 (4.57)	81	25.85 (19.99)	0.14 (0.07)	0.43
MZ II	1,567	251	6.16 (3.74)	75	20.89 (13.45)	0.21 (0.10)	0.56
MZ III	558	174	3.89 (3.79)	29	19.24 (13.97)	0.36 (0.12)	0.66
MZ IV	1,507	483	3.60 (3.24)	67	22.49 (13.88)	0.15 (0.07)	0.48
MZ V	282	65	4.81 (9.36)	15	18.80 (13.05)	0.22 (0.09)	0.52

### 2.2 Derivation of microsatellite genotypes for unique individuals

Genetic analysis was conducted at two molecular biological laboratories: the Molecular Ecology Lab at the U.S. Geological Survey Fort Collins Science Center (hereafter, FORT) and the National Genomics Center for Wildlife and Fish Conservation at the USFS Rocky Mountain Research Station (hereafter, NGC). DNA was extracted from feather and blood using previously described methods (Cross, Naugle, Carlson, & Schwartz, 2016; Row et al., 2015). Both FORT and NGC amplified 15 microsatellite loci in eight multiplex polymerase chain reactions (PCR) and genotypes were determined using electrophoresis (Cross et al., 2016; Row et al., 2015). Full genetic methods can also be found in Appendix S1.

Feather DNA samples can have low‐quality and quantity DNA. Therefore, to ensure correct genotypes from feather samples, each sample was PCR amplified at least twice across the 15 microsatellite loci to screen for allele dropout, stutter artifacts, and false alleles. Alleles for each locus were coded as missing if they did not match across at least two independent runs. Samples with missing genotypes for more than five loci were removed. Genotypes were then screened to ensure consistency between allele length and length of the microsatellite repeat motif. We used program DROPOUT v2.3 (McKelvey & Schwartz, 2005) and package ALLELEMATCH v2.5 (Galpern, Manseau, Hettinga, Smith, & Wilson, 2012) in R (R Core Team, 2016) to screen for genotyping error and to identify and remove multiple captures of the same individual.

To combine the genotype data sets from both labs, we first genotyped the same 70 individuals. Each laboratory's genotypes for these individuals were compared, and shifts in allele size were implemented to synchronize allele calls for all samples where necessary. Following the combination of samples, an additional ALLELEMATCH analysis was performed on the complete, combined data. Finally, we quantified the power of our microsatellite locus panel to discern individuals using probability identity (P_ID_; Evett & Weir, 1998) which calculates the probability that two individuals drawn at random from the population could have the same genotype across all loci.

### 2.3 Genetic differentiation within management zones

Sage‐grouse have clustered distributions (Doherty et al., 2016), particularly during the breeding season when individuals attend centralized breeding leks where the majority of our samples were collected. Additionally, sage‐grouse can have substantial within‐ and interseasonal movement distances (Cross, Naugle, Carlson, & Schwartz, 2017; Fedy et al., 2012), so we expected little differentiation between spatially proximate leks and individual samples (Row et al., 2015). Thus, we used a clustered (i.e., “group‐based”) approach to evaluate genetic differentiation as this likely best represents the ecology of the species at the spatial scale of this study and the sampling scheme (i.e., multiple samples from each lek). We derived lek clusters by grouping spatially proximate sampling locations using hierarchical clustering (*hclust* function with *complete* method) in R (R Core Team, 2016). The algorithm iteratively joined each sampling location based on distance using the Lance–Williams dissimilarity formula (Legendre & Legendre, 2012). Subsequently, we used a cut distance to differentiate clusters separated by a distance >25 km, which represented the maximum average summer to winter movement distance for any population in Wyoming (Fedy et al., 2012). We used a minimum sample size of eight individuals per genetic cluster for all subsequent analyses.

We calculated pairwise genetic differentiation using Hedrick's $G_{\mathrm{ST}}^{\prime }$ (Hedrick, 2005) among our clustered groups. This measure of genetic differentiation was highly correlated with *G*_ST_ (mean = 0.98 ± 0.02 *SD*) and Jost's *D*_est_ (mean = 0.94 ± 0.02 *SD*) and avoids problems associated with nonstandardized measures of genetic differentiation (Jost, 2008; Meirmans & Hedrick, 2011).

### 2.4 Landscape resistance within management zones

We quantified pairwise effective resistance among lek clusters using circuit theory. Landscape surfaces were coded such that each pixel was assigned a value representing resistance to gene flow. Subsequently, we used the *gdistance* package in R to quantify pairwise resistance between two groups as the expected correlate to the amount of gene flow between two groups (i.e., higher pairwise resistance equates to lower expected movement; McRae, 2006; McRae & Beier, 2007). Pairwise effective resistance values are a function of the flow of current—representative of gene flow—and therefore incorporate geographic distance (McRae & Beier, 2007).

We generated pairwise effective resistances from landscape resistance surfaces using three published models representing accepted abiotic and biotic variables influential in sage‐grouse biology (Doherty et al., 2016; see below for specific predictors). For each resistance surface, we tested three threshold values above or below which the landscape is hypothesized to be resistant to grouse movements (Keller et al., 2015). In all cases, we used the thresholds to derive a binary resistance surface representing habitat and nonhabitat. For variables with a known negative association with sage‐grouse (e.g., human disturbance, tillage), raster pixel values below the thresholds were set as habitat and assigned a resistance of 1 with nonhabitat cells receiving a higher value. When the landscape variable represented a positive association with sage‐grouse (e.g., percentage sagebrush cover, habitat utilization), values above the threshold were classified as habitat (resistance of 1) and we assigned higher values to nonhabitat (below the threshold). By determining the threshold values that best describe functional connectivity, we can make specific predictions as to when landscape degradation will lead to decreased connectivity. Below, we describe each resistance surface and their associated resistance and threshold values. All raster surfaces were resampled using bilinear interpolation to a resolution of 1.2 km. Overall, changes in resolution should not have a strong effect on pairwise resistance values (McRae & Beier, 2007; Row et al., 2015). In all cases, we compare results to resistances derived from an undifferentiated landscape in which all cells had a resistance of one. Thus, only distance was considered in the derivation of resistances, which serves as a null model and is analogous to geographic distances, but limited to the same study area constraints as the resistances surfaces (Lee‐Yaw, Davidson, McRae, & Green, 2009). Hereafter, we refer to these resistances as “geographic distances.”

#### 2.4.1 Development of habitat predictors

Our first set of resistance surfaces were built on a breeding habitat utilization model (BH) developed from landscape characteristics surrounding active breeding leks. The dominant variables varied for each MZ, but primarily included positive relationships with percent aerial coverage of sagebrush and negative relationships with increasing tree canopy cover and anthropogenic variables such as cultivation and human disturbance (Table 2). Details on the development of the BH models and top variables for each MZ are presented in Doherty et al. (2016). Raster values associated with the BH model represent the predicted probability that the raster pixel contains sufficient breeding habitat to support sage‐grouse lek formation. All active leks within the sage‐grouse range occurred in areas with a predicted probability (*p*) ≥ .65, so we used this as one of three thresholds, classifying all raster cell values with *p *≥* *.65 as habitat and anything below as nonhabitat and, as such, resistant to dispersal (Table 2). We expected that not all nonlek habitat (e.g., *p *<* *.65) would reduce dispersal. Thus, we considered two lower probability thresholds (*p *≥* *.5 and *p *≥* *.25) which split the distribution and in which all cells below the thresholds in *p* were assigned as nonhabitat to represent increased resistance to movement.

**Table 2:** Landscape variables used as resistance surfaces and associated thresholds used to delineate “habitat” and “nonhabitat” and the top selected resistance values. Additional details on habitat layers can be found in Doherty et al. (2016)

Description	Abbrev	Native resolution (m)	Expected effect	Thresholds	Resistances
Breeding habitat utilization: Range‐wide breeding habitat index developed independently for each management zone (Doherty et al., 2016)	BH	120 × 120	Positive	0.25, 0.50, 0.65	20, 200
Lek abundance quantified using kernel density estimation of lek counts in each management zone (Doherty et al., 2016).	KI	120 × 120	Positive	90%, 70%, 50%	10, 200
Breeding Population Index model combing breeding habitat utilization and lek abundance (BHU * KI; Doherty et al., 2016)	BPI	120 × 120	Positive	90%, 70%, 50%	10, 200
Sagebrush cover: Mean percentage of all sagebrush species (LANDFIRE EVT 1.2 – 2010) within 6.44 km moving windows	sb	30 × 30	Positive	10%, 30%, 50%	5, 50
Canopy cover: mean percent cover of the total tree canopy (LANDFIRE Fuels 1.2 – 2010) within 6.44 km moving windows	cc	30 × 30	Negative	5%, 10% 15%	5, 200
Tilled agricultural: mean percentage of tilled agricultural fields within 6.44 km moving windows. National Agriculture Statistics Service 2008–2014	ti	30 × 30	Negative	5%, 15%, 25%	5, 50
Human disturbance: index to human disturbance on the landscape including population density, roads, energy development. 2011 National Landcover Database Disturbed Classes (Homer et al., 2015).	hd	30 × 30	Negative	0.03%, 0.06%, 0.09%	5, 50
Steepness: Mean percentage of landscape classified as steep using Theobald LCAP tool. National Elevation Data (NED 2013, Data available from the U.S. Geological Survey)	st	30 × 30	Negative	5%, 10%, 15%	5, 200
Roughness: Standard deviation of elevation and averaged within 6.44 km moving windows (NED 2013)	ro	30 × 30	Negative	50, 100,150	5, 50
Annual Drought Index: Averaged across years and within 6.44 km moving windows estimated from USFS (1961–1990)	adi	1 km × 1 km	Negative	6,7,8	5, 50
Degree days above 5 C: The number of degrees that mean daily temperature is ≥5°C and averaged within 6.44 km moving windows	dd5	1 km × 1 km	Positive or Negative	1,600, 1,850, 2,050	5, 50

We also derived a set of resistance layers based on a kernel index (KI) of lek sizes and a breeding population index (BPI) model that combined estimates of breeding habitat (BH) and lek abundance (KI) estimates which represents areas that have both high‐quality breeding habitat and high lek abundances (KI * BH; Doherty et al., 2016). The distributions of predicted values for the KI and BPI were highly skewed toward zero. Thus, we used equally spaced percentiles (top 90%, 70%, and 50% of the BPI and KI) as our thresholds. The landmass represented by the BPI thresholds represented approximately 45%, 20% and 10% of the active sage‐grouse range included as habitat.

#### 2.4.2 Development of landscape predictors

We used eight landscape variables to develop landscape resistance surfaces independent of breeding habitat and abundance (Table 2). We used a subset of the variables that proved most important in Doherty et al. (2016) and which have previously been shown to influence sage‐grouse habitat. Landscape predictors included features that could be managed through protection (e.g., percent sagebrush cover) and restoration (e.g., canopy cover, tilled agriculture) and those that would be impossible to manage but have existed as barriers or which have restricted movement for a longer period of time (e.g., terrain topology, measured by steepness and roughness). Environmental conditions can also limit dispersal (Row et al., 2012). Thus, we also included two relevant environmental predictors: Annual Drought Index and degree days above 5°C (Doherty et al., 2016). In both cases, thresholds were primarily derived based on the distribution of values and values that split the distribution of values into evenly spaced bins to minimize correlation between resistance distances within a predictor (Table 2).

#### 2.4.3 Resistance values of nonhabitat

It is difficult to differentiate among a set of resistance surfaces that represent hypotheses of functional connectivity if they are highly correlated with each other or with distance (Row et al., 2017). To reduce correlations, we tested a range of resistances and choose values that would result in pairwise resistances that were not correlated with each other after increasing resistances. The resulting values were not the same for each surface. Thus, for all resistance surfaces, we tested increasing resistance values for nonhabitat (5, 10, and 20), and we calculated the correlation between the resulting pairwise resistance matrices and distance after each iteration. We chose our first nonhabitat resistance value when the average correlation (*r*) for the three derived resistance matrices (i.e., one for each threshold) was ≤0.7 for at least one of the MZs. We used a similar approach to choose a second resistance value by considering a new set of resistance values (50, 100, and 200) and selected the value when correlations averaged ≤0.7 from the first set for any MZ. Overall, this approach generated six resistance surfaces (3 thresholds * 2 resistance values) for each variable.

### 2.5 Within‐variable resistance thresholds

We identified the top resistance surfaces using maximum‐likelihood population‐effects models (MLPE), which is a mixed modeling approach that accounts for nonindependence in pairwise datasets (Clarke, Rothery, & Raybould, 2002; Van Strien et al., 2012). For each of the individual habitat, abundance, and landscape predictors, we used two analyses for each MZ to determine (i) the thresholds and resistance values that produced resistance distances (RD) that were best correlated with genetic differentiation, and (ii) whether that correlation was significantly greater than its correlation with distance. We determined the top model with Akaike's information criteria (AIC; Akaike, 1973) and estimated relative support using ΔAIC values compared with a model including only distance (i.e., the null model). We also determined the significance of standardized coefficients for the underlying model (GenDist ~ RD1) and a model that also includes geographic distance (GenDist ~ RD1 + RD_distance_). When the resistance coefficient confidence intervals were positive in both models, we considered the variable to have had a significant effect on gene flow over‐and‐above distance alone (Row et al., 2017).

### 2.6 Relative importance of landscape and habitat predictors

Within each MZ, we compared all of the models with significant resistance variables against each other using ΔAIC in a between‐variable analysis. There is some difficulty in identifying the correct multivariate models when resistance values are at different scales and have different distributions, but these results can be improved by calculating resistances from single landscapes that summarize multiple variables (Row et al., 2017). Therefore, in addition to comparing the univariate landscape predictors, we developed a combined landscape surface using all of the significant landscape predictors summed together. Cells with increased resistance for more than one landscape variable had an additive resistance value. We calculated pairwise resistances using this new combined additive surface and compared the fit with genetic differentiation to the fit for values derived from individual landscape variables and to the retained habitat predictors for each MZ.

### 2.7 Resistance model validations

#### 2.7.1 Individual‐based genetic differentiation

Although using a population‐based evaluation is most appropriate for our species and sampling regime, using an individual‐based approach in landscape genetics may influence the resulting perceived influence of landscape features (Prunier et al., 2013). Therefore, as a first validation of our results, we repeated the between‐variable analysis using an individual‐based approach and compared the results. We calculated individual‐based genetic differentiation using Bray–Curtis percentage dissimilarity among an individual's alleles, which reflects more recent changes in genetic differentiation when compared to other measures of individual differentiation (Landguth et al., 2010).

#### 2.7.2 Cross‐validation of predictive $G_{\mathrm{ST}}^{\prime }$

We validated our top selected resistance surface for each MZ using a Monte Carlo cross‐validation approach within each MZ. For each of 100 iterations, we split the data set into a training and testing dataset. Using the training dataset (80% of the data), we developed an MLPE model predicting genetic differentiation from the best resistance surface (GenDist ~ RD1) and distance alone (GenDist ~ RD_distance_). Subsequently, we predicted genetic differentiation based on the model, and we performed a cross‐validation using the testing dataset (the remaining 20% of the data) by determining the absolute difference between the predicted and actual genetic differentiation for both models using 100 iterations. We grouped differences into predictions ranging across five increasing categories of genetic differentiation to evaluate the ability of the model to predict genetic differentiation across a range of values.

### 2.8 Range‐wide connectivity

We used Circuitscape 4.0.1 (McRae & Shah, 2009) to derive and map predicted dispersal range‐wide by quantifying gene flow among all grouped locations, using the top validated surface for each MZ. We treated each lek cluster as a node and calculated average overall flow using the all‐to‐one mode. Thus, gene flow was estimated for each lek cluster to all others sequentially and then averaged.

## 3 RESULTS

### 3.1 Genetic differentiation within management zones

The number of lek clusters within MZs ranged from 15 to 81, and the mean number of individuals sampled per lek cluster was relatively consistent: ranging from 18.8 to 25.9 (Table 1). MZ III had the highest mean and maximum $G_{\mathrm{ST}}^{\prime }$, while all the other MZs exhibited less differentiation among clusters (Table 1). As expected, genetic differentiation ($G_{\mathrm{ST}}^{\prime }$) was positively correlated with distance for all MZs (Figure S2).

### 3.2 Within‐variable resistance surface analyses

#### 3.2.1 Landscape resistance surfaces

Effects of changing thresholds and resistance values on pairwise resistance varied among the MZs (Table S1). Most of this variation was tied to the abundance of the landscape classified as habitat, which varied between each MZ (Table S1). When a given landscape variable was rare on the landscape (e.g., tillage in MZ III and MZ V), changing the threshold or resistance had little effect on the pairwise resistance, resulting in high correlations in resistance across the thresholds. In most cases, changes in thresholds had a greater effect on resulting pairwise resistances than changes in resistance values (Table S1).

**Figure 2:**
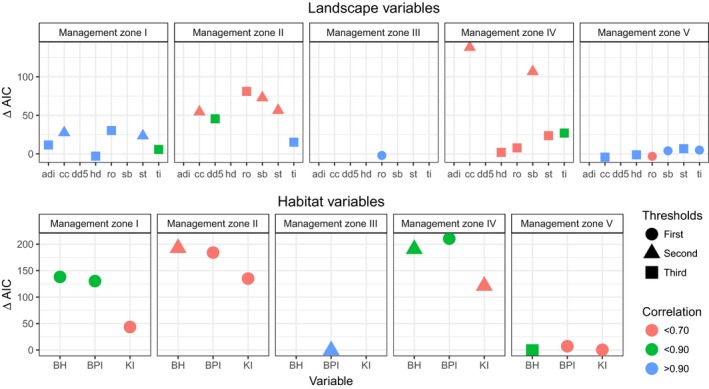
Top univariate coefficients for each landscape and habitat variable (see details in Table 2) in each management zone as compared to model fit with distances. Higher ∆ AIC values suggest a greater fit for the landscape surface as compared to distance alone. The top chosen threshold used to define habitat and nonhabitat and the level of correlation for resistances from that surface and distance are also shown

The importance of each landscape variable to functional connectivity varied across MZs. Terrain ruggedness was an important component of functional connectivity across the range as indicated by roughness (ro) and/or steepness (st) being significant for all MZs (Figure 2). Sagebrush cover was significant for functional connectivity in three of the five MZs with the selected threshold being either 10% (MZ V) or 30% (MZ II, IV) cover; values below these thresholds resulted in increased landscape resistance for “nonhabitat” (Figure 2). Sagebrush cover was not significant for functional connectivity in MZ I or MZ III, where mean sagebrush cover was the lowest (Table S1). Tree canopy cover reduced functional connectivity: >10% threshold had the highest ΔAIC value compared to geographic distance for MZs I, II, and IV. Changing thresholds or resistance values had little effect on pairwise resistance values in MZ V despite high canopy cover values that were clustered along MZ V boundaries.

Anthropogenic landscape disturbances also had consistent negative influences on functional connectivity. Human disturbance, tillage, or both human disturbance and tillage had significant effects on functional connectivity in all but MZ III (Figure 2). In MZ I, II, and IV, the thresholds for tillage were all 25% (Figure 2), suggesting disrupted functional connectivity on landscapes where row crops and small grain fields composed a greater percentage of the landscape than this threshold. In MZ V, the selected threshold was only 5%, but the overall percentage of the tilled agricultural in the landscape was also low (~2% on average; Table S1), and pairwise resistances were highly correlated (.99) with geographic distance. In MZ III, tilled agricultural similarly represented a small percentage of the landscape.

Landscape variables derived from environmental variables appeared to have little effect on functional connectivity for most MZs. Annual drought index (adi) was not significant for any MZs, and degree days above 5 (dd5) was only significant for MZ II (Figure 2).

#### 3.2.2 Breeding habitat utilization and abundance resistance surfaces

Similar to the landscape variables, the effects of changing thresholds and resistance values for habitat surfaces on pairwise resistances varied among the MZs, but was much greater for changes in thresholds overall (Table S1). In three of the five MZs, breeding habitat utilization (BH) improved model performance over undifferentiated landscapes, with ΔAIC values >100 when compared to geographic distance (Figure 2). The selected threshold for BH was either 0.25 (MZ I) or 0.5 (MZ II and IV), suggesting that when breeding habitat estimates dropped below these values in the respective zones, functional connectivity was reduced. In MZ IV, the correlation between resistances from a threshold of 0.25 and 0.50 was high (0.86), and the difference in model fit between the top two thresholds was less than the other two zones (ΔAIC = 10.42), suggesting the selection of the exact threshold may be less robust for this zone.

Univariate abundance alone (KI) performed better than distance in four of the five MZs, but ΔAIC values were not as high as BH in all of the MZs where they were both included in Figure 2. The combined abundance and breeding habitat layer (BPI) increased ΔAIC over an undifferentiated landscape and BH in MZ IV and MZ V. In MZ III, resistance values from an undifferentiated landscape had the lowest AIC, but the BPI model was similar with a small ΔAIC of 0.56 and the BPI coefficient confidence intervals in the model GEN ~ BPI + UNDIFF were significantly above zero (CI: 0.013–0.16).

### 3.3 Relative importance of landscape and habitat predictors

In four of the five MZs, resistance values derived from BH or BPI provided the best fit with genetic differentiation (Appendix S2). In MZ I and II, BH was the best model and ΔAIC values over the second‐best model were 7.93 and 8.50, respectively (Table 3). In both cases, BPI was the second‐best model. BPI was the best fit in MZ IV with a ΔAIC value of 19.83 over the second‐best model (BH), suggesting the incorporation of abundance significantly improved the model fit for this zone. In MZ III, the top model was an undifferentiated landscape, but as noted above, the second‐best model (BPI) had a low ΔAIC (0.56) and performed better in the prediction validation (see below). In MZ V, the combined landscape had the best fit, but again, the second‐best model was the BPI model with a relatively low ΔAIC of 2.61 (Table 3).

**Table 3:** Top models predicting pairwise genetic differentiation from pairwise resistance between lek groups of sage‐grouse samples. Resistance surface codes are a combination of the base predictor (Table 2), threshold value and assigned resistance

Surface	AICc	ΔAICc	AICc weight	Cumulative weight	Residual log‐likelihood
MZ I					
BH_25_020	−13,309.79	0.00	0.98	0.98	6,658.90
BPI_10_010	−13,301.86	7.93	0.02	1.00	6,654.94
landsum	−13,227.99	81.80	0.00	1.00	6,618.00
KI_10_020	−13,215.14	94.65	0.00	1.00	6,611.58
ro_150_005	−13,201.90	107.89	0.00	1.00	6,604.96
cc_10_005	−13,199.10	110.69	0.00	1.00	6,603.56
st_10_005	−13,194.98	114.81	0.00	1.00	6,601.50
adi_08_050	−13,183.16	126.63	0.00	1.00	6,595.59
ti_25_005	−13,177.29	132.51	0.00	1.00	6,592.65
distance	−13,171.60	138.19	0.00	1.00	6,589.81
hd_09_005	−13,168.67	141.12	0.00	1.00	6,588.34
MZ II					
BH_50_020	−10,116.80	0.00	0.99	0.99	5,062.41
BPI_10_010	−10,108.30	8.50	0.01	1.00	5,058.16
KI_10_020	−10,059.24	57.56	0.00	1.00	5,033.63
landsum	−10,059.15	57.65	0.00	1.00	5,033.58
ro_150_005	−10,005.27	111.53	0.00	1.00	5,006.64
sb_30_005	−9,996.98	119.82	0.00	1.00	5,002.50
st_10_005	−9,980.62	136.19	0.00	1.00	4,994.32
cc_10_005	−9,978.64	138.17	0.00	1.00	4,993.33
dd5_2050_005	−9,969.67	147.14	0.00	1.00	4,988.84
ti_25_050	−9,939.34	177.47	0.00	1.00	4,973.68
MZ III					
distance	−1,068.57	0.00	0.41	0.41	538.33
BPI_30_010	−1,068.00	0.56	0.31	0.71	538.05
landsum	−1,066.50	2.07	0.14	0.86	537.30
ro_050_005	−1,066.50	2.07	0.14	1.00	537.30
MZ IV					
BPI_10_010	−8,805.11	0.00	1.00	1.00	4,406.56
BH_50_020	−8,785.28	19.83	0.00	1.00	4,396.65
cc_10_005	−8,732.80	72.31	0.00	1.00	4,370.41
landsum	−8,724.33	80.78	0.00	1.00	4,366.17
KI_10_020	−8,715.78	89.33	0.00	1.00	4,361.90
sb_30_005	−8,701.29	103.83	0.00	1.00	4,354.65
ti_25_005	−8,621.57	183.54	0.00	1.00	4,314.80
st_15_005	−8,618.20	186.91	0.00	1.00	4,313.11
ro_150_005	−8,602.39	202.72	0.00	1.00	4,305.20
hd_09_005	−8,596.51	208.60	0.00	1.00	4,302.26
distance	−8,594.59	210.52	0.00	1.00	4,301.30
MZ V					
landsum	−335.76	0.00	0.60	0.60	172.08
BPI_50_010	−333.15	2.61	0.16	0.77	170.77
st_15_005	−332.72	3.04	0.13	0.90	170.56
ti_05_050	−330.89	4.87	0.05	0.95	169.65
sb_10_050	−329.96	5.80	0.03	0.98	169.18
KI_50_020	−326.44	9.32	0.01	0.99	167.42
distance	−325.98	9.78	0.00	0.99	167.19
BH_65_020	−324.98	10.78	0.00	1.00	166.69
hd_09_050	−324.77	10.99	0.00	1.00	166.58
ro_050_005	−322.80	12.96	0.00	1.00	165.60
cc_15_005	−321.68	14.08	0.00	1.00	165.04

### 3.4 Resistance model validations

#### 3.4.1 Individual‐based genetic differentiation

The individual‐based analysis produced results similar to the population‐based results presented above. Using the individual‐based analysis, BH or BPI was the top model in all zones with the exception of MZ V, where the summed landscape layer was the top surface as in the population‐based analysis (Table S2). In MZ I and MZ II, the order of the top two surfaces was switched from the population‐based analysis and BPI provided a better fit than BH. In MZ III, BPI provided a better fit over distance. In MZ IV, BH was the top surface over BPI—the best fit in the population‐based analysis—which was the second‐best fitting model.

#### 3.4.2 Cross‐validation of predictive $G_{\mathrm{ST}}^{\prime }$

**Figure 3:**
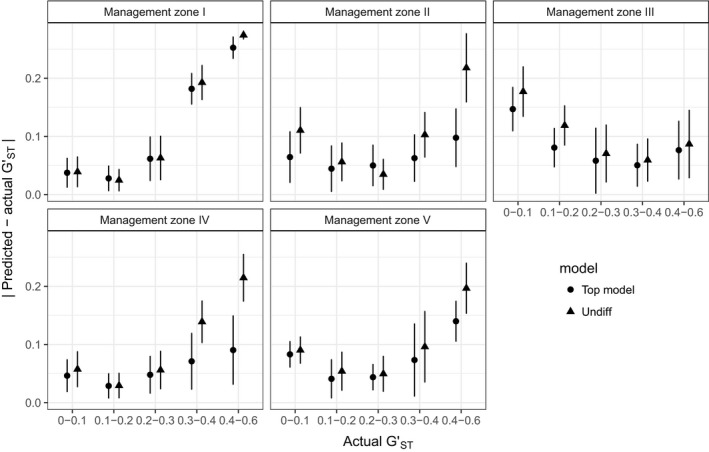
Absolute difference between predicted and actual pairwise genetic differentiation for groups of greater sage‐grouse samples with increasing levels of differentiation. Predictions for resistances from the top resistance model (see Table 3) and distance alone are compared

In all zones, the top resistance model was better able to predict $G_{\mathrm{ST}}^{\prime }$ than distance alone (Figure 3). The overall differences between the top model and geographic distance and the predictive ability of the top model varied among the MZs. In MZ I, IV and V, the top surface did not predict genetic differentiation better than geographic distance for clusters with low differentiation (<0.3 $G_{\mathrm{ST}}^{\prime }$), but had improved prediction for clusters with high differentiation (>0.3 $G_{\mathrm{ST}}^{\prime }$). In MZ II, the top model predicted $G_{\mathrm{ST}}^{\prime }$ better than distance for clusters with low and high $G_{\mathrm{ST}}^{\prime }$, but not for intermediate values of $G_{\mathrm{ST}}^{\prime }$ (Figure 3). In MZ III, the top surface only predicted $G_{\mathrm{ST}}^{\prime }$ better than geographic distance for clusters with low overall differentiation (<0.2 $G_{\mathrm{ST}}^{\prime }$).

### 3.5 Range‐wide patterns of gene flow

**Figure 4:**
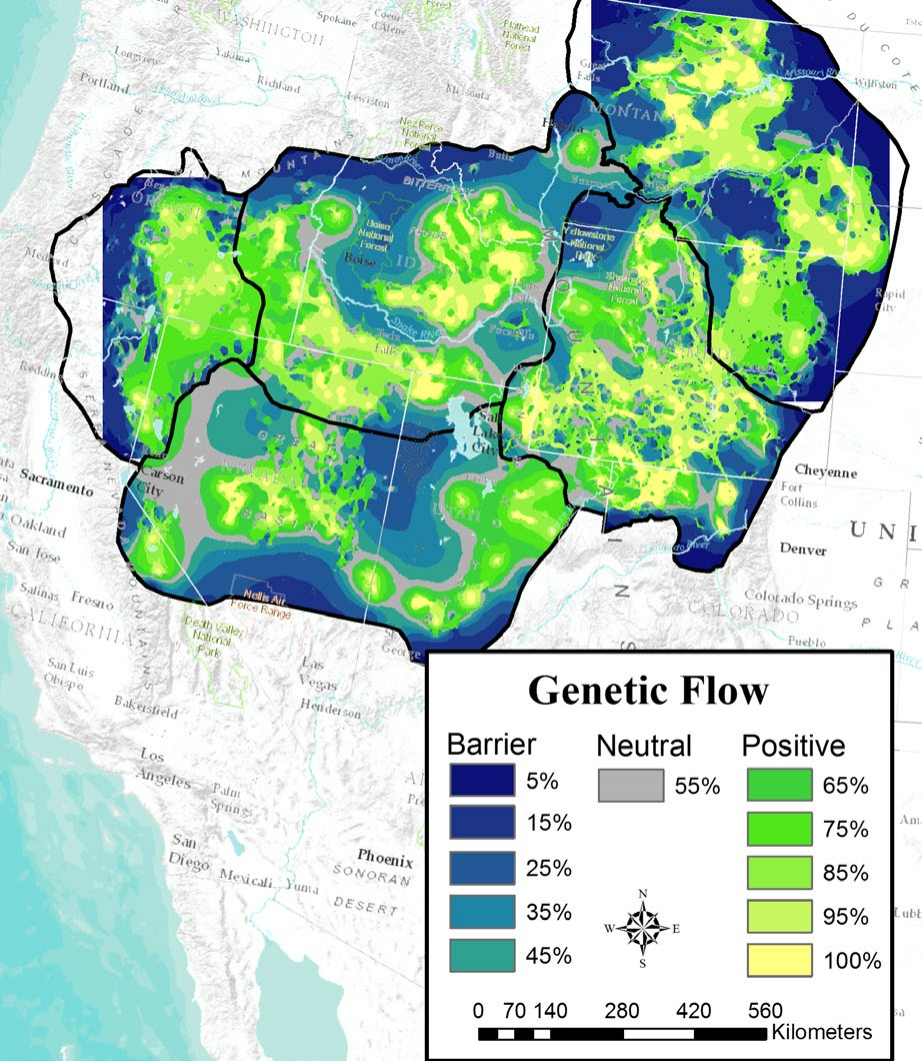
Range‐wide gene flow map describing low (dark blue) to high (yellow) connectivity between greater sage‐grouse lek groups used in landscape genetic analysis. For display flow was split into percentiles with bin labels representing the upper bound

Average gene flow between clusters identified connectivity between populations at local scales and at some regional scales within MZs (Figure 4). At the range‐wide scale, connectivity was more variable with large areas of low modeled gene flow separating some large population centers (Figure 4). Redundancies in high modeled gene flow throughout Wyoming and Colorado suggest that MZ II, which contains ~40% of range‐wide populations, is also the most connected of the five zones. Pathways of modeled gene flow suggest that eastern Montana and the Dakotas connect to MZ II via Wyoming's Powder River Basin in the southern extent of MZ I (Figure 4). North‐central Idaho and southwest Montana populations display ample gene flow between lek groups, but appear to lack regional connectivity to surrounding populations; mountainous terrain and intensive cultivation of Idaho's Snake River Plain are obvious barriers to gene flow. Similar breaks in modeled gene flow appear between MZ II and MZ III, but our current results highlight two potential paths of lowest resistance that bridge Rocky Mountain populations to those in the Great Basin. One that runs through the Three Creeks watershed near the town of Randolph in northeast Utah, and the other, a more southern loop of flow through central Utah. Farther west, multiple paths of potential gene flow connect southwest Idaho with Oregon and northeast California populations. On the California/Nevada border, two weak and diffuse paths connect the Bistate population to others to the north and east. Given that many of these paths span between management zones, more research is required in these regions to test the extent to which they represent areas of active gene flow.

## 4 DISCUSSION

Our landscape genetic analysis of gene flow and causal resistance surfaces represents one of the largest single evaluations of functional connectivity for a wild terrestrial vertebrate. Our primary advancements herein include accounting for regional variation in factors driving functional connectivity, deriving thresholds in functional connectivity for a species at risk, and depicting those responses as spatially explicit resistance surfaces for use in range‐wide conservation evaluations. Through noninvasive sampling techniques, the feasibility of developing broad‐scale range‐wide datasets is increasing. Furthermore, ever‐improving molecular genetic technologies are making it possible to generate large amounts of genotypic information from individuals in these datasets. Our findings can serve as a roadmap for other large‐scale evaluations in landscape genetics aimed at identifying connectivity thresholds and validating established resistance surfaces within regions of importance to wildlife management.

As hypothesized, MZ‐specific habitat metrics were the best predictors of differentiation. However, to our surprise, contributions of bird abundance‐corrected indices were equivocal to model fit. Collectively, these findings suggest that maintenance of breeding habitat above critical thresholds so as not to reduce genetic exchange is fundamental to conservation of connected populations. Our inferences also add to a body of literature suggesting that structural connectivity estimated from habitat indices can be a strong predictor of functional connectivity for many species (Hagerty, Nussear, Esque, & Tracy, 2011; Row et al., 2010, 2015; Shafer et al., 2012; Wang et al., 2008), but we stress the importance of including model validations to have a clear understanding of the predictive capabilities of landscape genetic models.

Herein, BH models in each zone were developed independently and the relative importance of the variables underlying each breeding habitat model varied. Broadly speaking, the variables with the greatest importance were a positive association with sagebrush cover and a negative association with increased tree canopy cover for most zones. When resistance surfaces from these landscape variables were compared independently to genetic differentiation, they were similarly found have a greater fit than distance alone, demonstrating their overall importance. Interestingly, the distribution of Annual Drought Index (MZ II, MZ IV, MZ V) and degree days >5°C (MZ II, MZ III, MZ IV) were prominent variables in some of the habitat models, but when analyzed independently they had little explanatory power in our assessment of functional connectivity. Thus, their importance may be connected or correlated with other variables.

Using our threshold approaches, we found that landscapes with a probability of occurrence for breeding leks <0.25 or 0.5 reduced functional connectivity. These values are likely below the threshold for persistence, as all known active breeding leks are present in regions with values >0.65 (Doherty et al., 2016). The lower value for functional connectivity is not surprising, as individuals are often willing to disperse through undesirable habitat (Tischendorf & Fahrig, 2000), but has important conservation implications on how to manage landscapes to preserve functional connectivity for sage‐grouse. For example, although the habitat may be degraded below the 0.65 threshold for breeding lek formation, it will still be important to maintain habitat above the lower thresholds identified here in order to maintain functional connectivity.

It is important to point out that landscape genetic results can vary at different spatial scales (Anderson et al., 2010) and habitat configuration can influence both dispersal (D'Eon, Glenn, Parfitt, & Fortin, 2002) and habitat selection (Wisdom, Meinke, Knick, & Schroeder, 2011). Furthermore, different threshold values had different overall effects on pairwise resistance and on model fit for each management zone. Thus, in planning for conservation, conducting analyses at different spatial extents and testing the effect of the different thresholds identified here (0.5, 0.25) and the resulting habitat configuration will provide valuable insights.

How individuals respond to habitat structure will vary between species. Thus, comparing other species that overlap our study area, especially other sagebrush obligate species, could provide insight into the generality of the results found here. We are not aware of other studies that have utilized a threshold approach. However, both Wang et al. (2008) and Row et al. (2010) found that the best fitting resistance models assigned very low‐quality habitat disproportionately higher resistances or set these habitats as absolute barriers. These results suggest that thresholds in habitat indices may be an effective approach for other species. Yet, in some cases, continuous habitat surfaces have performed better than discrete values (Hagerty et al., 2011), or individual landscape predictors have performed better than combined habitat indices altogether (Roffler et al., 2016; Wasserman, Cushman, Schwartz, & Wallin, 2010). A weak association between habitat and functional connectivity is likely when there are large differences between daily use and dispersal habitat or when one or a few landscape components are the primary drivers of functional connectivity. It is also hard to compare between studies when different approaches are used to derive resistance surfaces. For example, both Wasserman et al. (2010) and Roffler et al. (2016) only tested habitat resistance surfaces with a direct linear relationship between habitat and landscape resistance. In contrast, discrete values (tested here and by Wang et al., 2008 and by Row et al., 2010) or the exponential relationships between habitat and resistance (Row et al., 2015) often appear to provide better model fit.

We predicted that the inclusion of abundance information would improve the fit between habitat and genetic differentiation. Local population size has long been linked to patterns of dispersal and can influence source‐sink dynamics (Matthysen, 2005; Ozgul, Oli, Armitage, Blumstein, & Van Vuren, 2009). Furthermore, population size will influence the effects of genetic drift and have impacts on spatial patterns of genetic differentiation (Hutchison & Templeton, 1999). However, contrary to our predictions including abundance information into the resistance surfaces did not improve the model fit for most MZs. In our analysis, inclusion of abundance indices acted to decrease resistance around population centers and increase the resistance in areas with low abundance, but our approach could not test the effect of population size on the attractiveness of individual locations to dispersers. Thus, using models where local abundance can modify the number of dispersers into, or out of, a population (Murphy, Dezzani, Pilliod, & Storfer, 2010; Row, Oyler‐McCance, & Fedy, 2016), might provide more insight into the effects of abundance on differentiation. We only included abundance estimates of males during the breeding season, but it is possible that abundance during other seasons or estimates of effective population size may be of greater importance to functional connectivity.

### 4.1 Resistance thresholds and spatial variation in landscape predictors

Terrain roughness shaped functional connectivity across all MZs, and sagebrush availability (+) or tree canopy cover (−), and extent of cultivation (−) were influential in all but MZ III, the most highly fragmented zone. Time lags exist from when a barrier to dispersal first arises on the landscape and when the influence of that barrier on functional connectivity can be detected (Landguth et al., 2010). For sage‐grouse, the topography of the terrain can have a strong influence on habitat use and distribution (Davis, Reese, Gardner, & Bird, 2015; Fedy et al., 2014), and these imposed restrictions to movement or barriers have likely remained static for millennia. Indeed, in other large‐scale landscape genetic evaluations of sage‐grouse, areas with higher landscape ruggedness, a measure of sharp changes in elevation, restricted gene flow (Row et al., 2015). Similarly, in our individual landscape analysis, we found increases in steepness or roughness reduced functional connectivity in all of the five MZs, likely resulting from avoidance of these areas by dispersing sage‐grouse. Although these are natural landscape features and will not be the target of conservation initiatives, it is clear that they impact dispersal and so it is important to consider and control for terrain in future evaluations.

In contrast to terrain topography, the current distribution of sagebrush has been modified through development and land conversion (Davies et al., 2011; Welch, 2005). Thus, many resistance features have likely been created or modified since human settlement. Sage‐grouse rely on sagebrush for both food and shelter and sagebrush is a strong predictor of sage‐grouse habitat across seasons and spatial scales (Connelly, Knick, Schroeder, & Stiver, 2004; Doherty et al., 2016; Fedy et al., 2014), and it influences functional connectivity (Row et al., 2015). Similarly, we found that areas with low sagebrush cover impeded gene flow in three of the five MZs. In two cases, we found that sagebrush cover <30% impacted dispersal, with a 10% threshold in the third, suggesting that sagebrush cover <10%–30% reduces gene flow. As with the habitat thresholds, sage‐grouse are capable of dispersal through habitats in which they are unlikely to persist (suggested persistence thresholds are in the range of 40%–65% sagebrush cover; Aldridge, Nielsen, & Boyce, 2008; Wisdom et al., 2011; Knick, Hanser, & Preston, 2013).

Given the importance of sagebrush to sage‐grouse, it is surprising that the distribution of sagebrush cover was not a significant predictor of genetic differentiation in two of the MZs. Variation in the importance of a predictor can be related to its abundance and distribution, but this does not seem to be the case here, as mean values were similar across all zones. In MZ I, where individuals have been shown to move far distances (Cross et al., 2017; Newton et al., 2017), isolation by distance appeared to drive differentiation. None of the landscape predictors were very strong in MZ III, a zonal boundary spanning multiple populations and/or habitat–population relationships. Perhaps this should be expected as MZ III is set in basin and range topography with this natural fragmentation exacerbated by conifer encroachment and land‐use change (Chambers et al., 2017). In both of these zones, sagebrush was an important component of the derived habitat indices suggesting that its distribution is important in combinations with other landscape variables.

A long history of fire control has enabled encroaching conifer woodlands to degrade sagebrush habitats into areas with higher amounts of tree canopy cover. Sage‐grouse avoid canopy cover at low levels (<4%, Miller, Naugle, Maestas, Hagen, & Hall, 2017) or stay and suffer demographic impacts (Coates et al., 2017). In conifer removal areas, females readily nested in restored sites (Coates et al., 2017; Severson et al., 2017) and were more successful in raising their broods (Sandford et al., 2017). We build on this knowledge to add that connectivity among population centers is reduced when conifer expansion exceeds a 10% threshold in canopy cover. Connectivity is relevant to management because conifer‐encroached habitats stimulate faster yet riskier movements, especially in juveniles, that may make sage‐grouse more vulnerable to visually acute predators with demonstrated fitness consequences (Prochazka et al., 2017). Future restoration planning with the goal of improving genetic connectivity can use our range‐wide resistance surfaces to select areas where the greatest benefit may be found.

Cultivation is known to reduce breeding populations (Doherty et al., 2016; Smith et al., 2016). Findings here further suggest that cultivation reduces gene flow when >25% of the landscape is converted to cropland in three of five MZs tested. In eastern Montana, where cultivation is most prevalent, 70% of the best sage‐grouse habitat is privately owned. Therefore, activities that keep sagebrush habitats intact (such as large working cattle ranches) as opposed to those that do not (such as agriculture) should help maintain connectivity between population strongholds. Cultivation land use was not prevalent in the other two zones (MZ III, V) so we had low power to determine its effects on functional connectivity therein.

### 4.2 Resistance model validation

Landscape genetic studies typically quantify correlations between patterns of genetic differentiation or gene flow with landscape resistance (or cost) distances and provide insight into the relative importance of landscape variables influencing functional connectivity (Manel et al., 2003). Here, we not only evaluated these common objectives, but also tested the ability of the top linear mixed models to predict genetic differentiation among populations based on landscape resistance. Predictive ability of our resistance maps varied quite dramatically among MZs and also with the level of differentiation between the populations considered. In most cases, improvement in predictive ability of our models when compared to the null distance model was greatest when pairwise comparisons had little (MZ III) or much (MZ IV, MZ V, MZ I) differentiation among populations and in one case where differentiation among populations varied (MZ II). Where our models did not perform better, results indicated that distance was the predominate driver of differentiation and that our models did not add much predictive improvement. For example, in MZ IV, plots of genetic differentiation and distance (Figure S2) reveal that highly differentiated populations are not explained well by distance alone due to the high value of residuals; the resistance models for this zone have much better predictive power for these highly differentiated groups. Conversely, in MZ III, the residuals are larger for populations with low differentiation, which corresponds to the improved performance of the resistance models.

Comparing performance of our models to other studies is not possible as we are unaware of others who have validated the predictive power of their landscape resistance models. The potential power of landscape genetics for informing management will be greatly improved if the model results can be used to predict genetic differentiation between regions where genetic data are lacking or to predict how changes to the landscape are likely to impact functional connectivity. Predictions backed by any confidence require more validation than is current practice, and we present a novel framework for providing this necessary model validation.

### 4.3 Implications for sage‐grouse management

Currently, sage‐grouse conservation is largely focused on implementing beneficial conservation measures within population strongholds (e.g., Priority Areas of Conservation; U.S. Fish and Wildlife Service 2013) and around known sage‐grouse leks as they represent areas of importance for breeding and early brood rearing habitat (Doherty et al., 2016; Fedy et al., 2014). However, our analyses and resulting resistance surfaces point to several measures that can be taken to help improve and maintain functional connectivity for sage‐grouse. First, although population strongholds likely have much higher suitability values, maintaining areas outside of these regions above habitat thresholds of 0.5, or potentially 0.25 in some management zones, should help maintain connectivity between these existing protection areas. Secondly, our models could help identify landscapes where targeted conservation would maximize conservation return on investment. For example, a 100‐year history of fire suppression has enabled conifer expansion into sagebrush habitats, reducing lek attendance, breeding habitat quality and survival of sage‐grouse (Coates et al., 2017; Miller et al., 2017). We found that functional connectivity appeared to drop at around 10% and, thus, a conifer removal strategy incorporating known dispersal pathways from our current flow map (Figure 4) may help to maintain connectivity between population strongholds.

In addition to the thresholds we identified, the resistance surfaces and gene flow maps we generated help identify areas within which to prioritize management actions. Resistance maps identify areas that are above and below threshold values that obstruct gene flow and direct conservation actions within these areas where it is possible to maintain or improve habitat above or below a targeted threshold. Furthermore, because the nodes in our analysis represent clusters of active sage‐grouse leks, the modeled gene flow should reflect movement from these high density areas and, as such, can be used to help locate and protect dispersal corridors. Additional gene flow maps can be produced among management areas of particular interest to managers and used to target conservation initiatives that will maintain connectivity among population strongholds.

It was clear from our cross‐validation that the predictive ability of our resistance models varied with the levels of genetic differentiation and among management zones. Even when our results strongly suggested an improvement in model fit when compared to the null distance model, the overall predictive ability of our models was at times marginal or poor depending on the amount of genetic differentiation among populations. Without our cross‐validation to provide an estimate of predictive ability, conservation initiatives could direct actions that will not have the desired improvement on connectivity. Overall, our cross‐validated approach used in developing our threshold resistance surfaces for sage‐grouse should initiate a new era of spatial analyses which emphasizes the value of functional connectivity and the identification of habitats supporting it.

## DATA ARCHIVING STATEMENT

Cluster‐based genotypic data used for the analysis in this article are available on the USGS ScienceBase website: https://www.sciencebase.gov/ (https://doi.org/10.5066/f7rb73v0).

## CONFLICT OF INTEREST

None declared.
