# Supplementary material for: Quantifying functional connectivity: The role of breeding habitat, abundance, and landscape features on range‐wide gene flow in sage‐grouse
Source: Evol Appl. 2018 May 12;11(8):1305–21. doi: 10.1111/eva.12627 (PMC6099827; doi:10.1111/eva.12627)
Supplement: Supplementary file 1 [file EVA-11-1305-s001.docx]

**Figure S1.** Locations of 6,844 greater sage-grouse samples (black crosses) collected from 2005 to 2014 and groups (red circles) used to compare genetic differentiation and landscape resistance. Shaded gray area represents the current distribution of greater sage-grouse and dotted lines represent the extent of seven management zones for the species.

**Figure S2.** Correlation between genetic distance (G’_ST_) and geographic distance (i.e., resistance on the x-axis). To be consistent with the resistance surfaces, we estimated distance by calculating the resistances using circuit theory for all pairwise cluster comparisons across an undifferentiated landscape. Results are presented for each management zone. Each grey dot represents pairwise distance and resistance between two groups and the red line is linear of best fit estimated with a linear model.
