# Supplementary material for: Quantifying functional connectivity: The role of breeding habitat, abundance, and landscape features on range‐wide gene flow in sage‐grouse
Source: Evol Appl. 2018 May 12;11(8):1305–21. doi: 10.1111/eva.12627 (PMC6099827; doi:10.1111/eva.12627)
Supplement: Supplementary file 2 [file EVA-11-1305-s002.docx]

**Table S1.** Effects of changing thresholds and resistance for each landscape layer in each management zone. Mean correlations for 1) all resistance surfaces (Table1) and resistances derived from an undifferentiated landscape, 2) mean correlations between resistance surfaces with the same resistance value but different threshold, and 3) mean correlations between surfaces with same resistance value, but different landscape threshold. The mean raster value representing the mean amount of the variable present in different management zones.

| **Management Zone** | **Mean correlation with UNDIFF** | **Mean correlation between different thresholds (same resistance)** | **Mean correlation between different resistances (same threshold)** | **Mean raster value** |
| --- | --- | --- | --- | --- |
|  |  | Breeding habitat (BH) |  |  |
| MZ I | 0.56 | 0.72 | 0.96 | 0.44 |
| MZ II | 0.51 | 0.73 | 0.94 | 0.51 |
| MZ III | 0.82 | 0.89 | 0.98 | 0.45 |
| MZ IV | 0.58 | 0.81 | 0.97 | 0.45 |
| MZ V | 0.61 | 0.69 | 0.95 | 0.37 |
|  |  | Abundance (KI) |  |  |
| MZ I | 0.60 | 0.69 | 0.99 | 0.03 |
| MZ II | 0.53 | 0.69 | 0.99 | 0.07 |
| MZ III | 0.80 | 0.77 | 0.99 | 0.04 |
| MZ IV | 0.59 | 0.70 | 0.99 | 0.05 |
| MZ V | 0.53 | 0.76 | 0.97 | 0.08 |
|  |  | BPI |  |  |
| MZ I | 0.63 | 0.72 | 0.97 | 0.02 |
| MZ II | 0.56 | 0.71 | 0.97 | 0.05 |
| MZ III | 0.81 | 0.79 | 0.98 | 0.03 |
| MZ IV | 0.64 | 0.72 | 0.97 | 0.03 |
| MZ V | 0.65 | 0.85 | 0.92 | 0.05 |
|  |  | Sagebrush cover (sb) |  |  |
| MZ I | 0.45 | 0.60 | 0.97 | 21% |
| MZ II | 0.43 | 0.73 | 0.89 | 31% |
| MZ III | 0.58 | 0.71 | 0.92 | 28% |
| MZ IV | 0.54 | 0.66 | 0.93 | 30% |
| MZ V | 0.59 | 0.60 | 0.91 | 42% |
|  |  | Tree canopy closure (cc) |  |  |
| MZ I | 0.82 | 0.68 | 0.88 | 4.19% |
| MZ II | 0.41 | 0.86 | 0.88 | 9.6% |
| MZ III | 0.36 | 0.81 | 0.93 |  |
| MZ IV | 0.64 | 0.90 | 0.77 | 8.77% |
| MZ V | 0.96 | 0.99 | 0.99 | 7.58% |
|  |  | Human disturbance (hd) |  |  |
| MZ I | 0.60 | 0.57 | 0.94 | 0.05 |
| MZ II | 0.51 | 0.43 | 0.89 | 0.05 |
| MZ III | 0.40 | 0.51 | 0.86 | 0.05 |
| MZ IV | 0.25 | 0.47 | 0.93 | 0.05 |
| MZ V | 0.49 | 0.43 | 0.95 | 0.05 |
|  |  | Tillage (ti) |  |  |
| MZ I | 0.34 | 0.63 | 0.83 | 10 % |
| MZ II | 0.79 | 0.70 | 0.96 | 3 % |
| MZ III | 0.98 | 0.98 | 0.99 | 2 % |
| MZ IV | 0.48 | 0.61 | 0.88 | 6 % |
| MZ V | 1.00 | 1.00 | 1.00 | 2 % |
|  |  | Roughness (ro) |  |  |
| MZ I | 0.63 | 0.84 | 0.84 | 809.51 |
| MZ II | 0.48 | 0.68 | 0.93 | 759.27 |
| MZ III | 0.66 | 0.65 | 0.93 | 734.14 |
| MZ IV | 0.28 | 0.67 | 0.94 | 735.30 |
| MZ V | 0.50 | 0.71 | 0.95 | 758.07 |
|  |  | Steepness (st) |  |  |
| MZ I | 0.93 | 0.93 | 0.98 | 4 % |
| MZ II | 0.39 | 0.71 | 0.82 | 10 % |
| MZ III | 0.39 | 0.82 | 0.90 | 11 % |
| MZ IV | 0.23 | 0.79 | 0.87 | 14 % |
| MZ V | 0.49 | 0.80 | 0.92 | 5 % |
|  |  | Degree days above 5 (dd5) |  |  |
| MZ I | 0.52 | 0.52 | 0.96 | 2408.87 |
| MZ II | 0.36 | 0.52 | 0.90 | 2735.90 |
| MZ III | 0.43 | 0.70 | 0.91 | 2207.96 |
| MZ IV | 0.26 | 0.54 | 0.95 | 2727.76 |
| MZ V | 0.52 | 0.73 | 0.92 | 2412.94 |
|  |  | Annual drought index (adi) |  |  |
| MZ I | 0.43 | 0.22 | 0.95 | 31.53 |
| MZ II | 0.20 | 0.64 | 0.93 | 32.21 |
| MZ III | 0.37 | 0.70 | 0.89 | 28.60 |
| MZ IV | 0.20 | 0.64 | 0.92 | 32.25 |
| MZ V | 0.57 | 0.90 | 0.92 | 30.26 |

**Table S2.** Top models predicting pairwise genetic distance (Bray-Curtis) from pairwise resistance between sage-grouse individuals. Resistance surface codes are a combination of the base predictor (Table 2), threshold value and assigned resistance.

| **Surface** | **AICc** | ΔAIC**c** | **AICc**  **Weight** | **Cumulative**  **Wegith** | **Residual**  **Log-Likelihood** |
| --- | --- | --- | --- | --- | --- |
| *Zone1* | | | | | |
| BPI_10_010 | -6679138 | 0 | 1 | 1 | 3339573 |
| BH_25_020 | -6677872 | 1265.65 | 0 | 1 | 3338940 |
| landsum | -6677707 | 1431.41 | 0 | 1 | 3338857 |
| st_10_005 | -6676851 | 2286.92 | 0 | 1 | 3338430 |
| adi_08_050 | -6676704 | 2433.66 | 0 | 1 | 3338356 |
| ro_150_005 | -6676951 | 2187.42 | 0 | 1 | 3338479 |
| hd_09_005 | -6676160 | 2977.91 | 0 | 1 | 3338084 |
| cc_10_005 | -6677258 | 1879.76 | 0 | 1 | 3338633 |
| ti_25_005 | -6677600 | 1537.83 | 0 | 1 | 3338804 |
| KI_10_020 | -6677361 | 1776.87 | 0 | 1 | 3338685 |
| undiff | -6676141 | 2996.63 | 0 | 1 | 3338075 |
| *Zone2* | | | | | |
| BPI_10_010 | -3721440 | 0 | 1 | 1 | 1860724 |
| BH_50_020 | -3721393 | 46.43 | 0 | 1 | 1860701 |
| landsum | -3721140 | 299.35 | 0 | 1 | 1860574 |
| sb_30_005 | -3720756 | 683.95 | 0 | 1 | 1860382 |
| KI_10_020 | -3720395 | 1044.77 | 0 | 1 | 1860201 |
| cc_10_005 | -3720349 | 1090.33 | 0 | 1 | 1860179 |
| ro_150_005 | -3720094 | 1345.57 | 0 | 1 | 1860051 |
| st_10_005 | -3719660 | 1779.6 | 0 | 1 | 1859834 |
| dd5_2050_005 | -3716605 | 4834.47 | 0 | 1 | 1858307 |
| ti_25_050 | -3716986 | 4453.24 | 0 | 1 | 1858497 |
| *Zone 3* | | | | | |
| BPI_30_010 | -601249.2 | 0 | 1 | 1 | 300628.6 |
| undiff | -600918.3 | 330.93 | 0 | 1 | 300463.2 |
| landsum | -600241.2 | 1007.97 | 0 | 1 | 300124.6 |
| ro_050_005.1 | -600241.2 | 1007.97 | 0 | 1 | 300124.6 |
| *Zone 4* | | | | | |
| BH_50_020 | -3910745 | 0 | 1 | 1 | 1955377 |
| cc_10_005 | -3910615 | 130.52 | 0 | 1 | 1955311 |
| BPI_10_010 | -3910331 | 414.02 | 0 | 1 | 1955170 |
| landsum | -3910059 | 686.48 | 0 | 1 | 1955033 |
| sb_30_005 | -3909798 | 947.58 | 0 | 1 | 1954903 |
| KI_10_020 | -3909258 | 1487.43 | 0 | 1 | 1954633 |
| st_15_005 | -3909184 | 1561.5 | 0 | 1 | 1954596 |
| ro_150_005 | -3909037 | 1708.08 | 0 | 1 | 1954523 |
| hd_09_005 | -3906954 | 3791.06 | 0 | 1 | 1953481 |
| ti_25_005 | -3907670 | 3075.26 | 0 | 1 | 1953839 |
| undiff | -3907447 | 3298.26 | 0 | 1 | 1953728 |
| *Zone 5* | | | | | |
| landsum | -159520.2 | 0 | 1 | 1 | 79764.1 |
| hd_09_050 | -159425.9 | 94.28 | 0 | 1 | 79716.96 |
| undiff | -159382.7 | 137.48 | 0 | 1 | 79695.37 |
| BPI_50_010 | -159378.3 | 141.91 | 0 | 1 | 79693.15 |
| cc_15_005 | -159374 | 146.17 | 0 | 1 | 79691.02 |
| st_15_005 | -159364.1 | 156.1 | 0 | 1 | 79686.06 |
| BH_65_020 | -159354.8 | 165.45 | 0 | 1 | 79681.38 |
| KI_50_020 | -159293.2 | 227.03 | 0 | 1 | 79650.59 |
| ro_050_005 | -159242.7 | 277.53 | 0 | 1 | 79625.34 |
| ti_05_050 | -159101 | 419.18 | 0 | 1 | 79554.52 |
| sb_10_050 | -158788.7 | 731.5 | 0 | 1 | 79398.35 |
