# Supplementary material for: Quantifying functional connectivity: The role of breeding habitat, abundance, and landscape features on range‐wide gene flow in sage‐grouse
Source: Evol Appl. 2018 May 12;11(8):1305–21. doi: 10.1111/eva.12627 (PMC6099827; doi:10.1111/eva.12627)
Supplement: Supplementary file 3 [file EVA-11-1305-s003.docx]

**Appendix A.** Detailed methods and materials for DNA extraction and genotyping.

*DNA extraction and ampliﬁcation*

Genetic analysis was conducted at two molecular biological laboratories; the Molecular Ecology Lab at the U.S. Geological Survey Fort Collins Science Center (hereafter, FORT) and the National Genomics Center for Wildlife and Fish Conservation at the USFS Rocky Mountain Research Station (hereafter, NGC). At NGC feather DNA was extracted from the quill (calamus) using QIAGEN’s DNeasy Blood and Tissue Kit and a user-developed protocol for puriﬁcation of total DNA from nails, hair, or feathers. We modiﬁed the protocol by incubating samples for a minimum of 8 h after addition of Proteinase K and by eluting DNA with 100 µl of Buffer AE (Cross et al. 2016). Feather samples from NGC were extracted in a lab used only for non-invasive DNA extraction in order to avoid potential contamination from samples with higher DNA concentrations. Blood samples were extracted using QIAGEN’s DNeasy Blood and Tissue Kit and the associated protocol for nucleated blood. At FORT, DNA from feather and blood samples were extracted using the same QIAGEN Kit following the user-developed protocol with portions of the extraction process automated using a Qiacube (QIAGEN).

Both FORT and NGC ampliﬁed 15 microsatellite loci and one sex-diagnostic locus (CDH gene, using the primers 1237L and 1272H; Kahn et al. 1998) in eight multiplex polymerase chain reactions (PCR). At FORT, PCR products were multi-loaded based on product size and primer label, combined with GeneScan LIZ 600 internal lane size standard (Applied Biosystems), and electrophoresed through a capillary gel matrix using an AB3500 Automated DNA Sequencer (Applied Biosystems). Allele sizes were determined for each locus using GeneMapper v4.1 software (Applied Biosystems) (for details see Row *et al.* 2015). NGC electrophoresis methods can be found in Cross *et al.* (2016).

*Genotyping*

Feather DNA samples can have low quality and quantity DNA. Therefore, to ensure correct genotypes from feather samples, each sample was PCR ampliﬁed at least twice across the 15 microsatellite loci to screen for allele dropout, stutter artifacts, and false alleles. Alleles for each locus were coded as missing if they did not match across at least two independent runs. Samples with missing data for more than 5 loci were removed. Genotypes were then screened to ensure consistency between allele length and length of the microsatellite repeat motif. We used program DROPOUT v2.3 (McKelvey and Schwartz 2005) and package ALLELEMATCH v2.5 (Galpern et al. 2012) in R (R Core Team 2016) to screen for genotyping error and to identify and remove multiple captures of the same individual.

To combine the genotype data sets from both labs, we first genotyped the same 70 individuals. For two loci, the labs had a two base pair difference across all allele calls (BG6 and SGCA5), for one locus there was a four base pair difference (SGCA11) and for another locus, a seven base pair difference (SGCTAT1) as well as two alleles called off-step on a dimer repeat motif changed to comply with the tetramer repeat motif. Each lab’s genotypes for these individuals were shifted to synchronize allele calls for all samples for these loci. Following the combination of samples, an additional ALLELEMATCH analysis was performed on the complete, combined, data. Finally, we quantiﬁed the power of our microsatellite locus panel to discern individuals using probability identity (P_ID_; Evett and Weir 1998) which calculates the probability that two individuals drawn at random from the population have the same genotype across all loci. P_ID_ for the complete microsatellite panel was 2.20x10^-22^, providing evidence that our microsatellite panel was adequate for distinguishing individuals.

**Literature Cited**

Cross, T. B., D. E. Naugle, J. C. Carlson, and M. K. Schwartz. 2016. Hierarchical population structure in greater sage-grouse provides insight into management boundary delineation. Conservation Genetics 17:1417–1433.

Evett, I., and B. S. (Bruce S. . Weir. 1998. Interpreting DNA evidence: statistical genetics for forensic scientists. Sinauer Associates, Sunderland, MA.

Row, J. R., S. J. Oyler-McCance, J. A. Fike, M. S. O’Donnell, K. E. Doherty, C. L. Aldridge, Z. H. Bowen, and B. C. Fedy. 2015. Landscape characteristics influencing the genetic structure of greater sage-grouse within the stronghold of their range: a holistic modeling approach. Ecology and Evolution 5:1955–1969.
