# Supplementary material for: Quantifying functional connectivity: The role of breeding habitat, abundance, and landscape features on range‐wide gene flow in sage‐grouse
Source: Evol Appl. 2018 May 12;11(8):1305–21. doi: 10.1111/eva.12627 (PMC6099827; doi:10.1111/eva.12627)
Supplement: Supplementary file 4 [file EVA-11-1305-s004.docx]

**Appendix B.** Spatial representation for top resistance surfaces for five sage-grouse management zones I-V.

**Figure B.1.** The best establish landscape resistance surface for MZI was the breeding habitat model (BH_25_20) where the predicted breeding habitat probabilities below 0.25 were designated as non-habitat and set to a resistance of 20. The breeding habitat model was comprised of a variety of landscape variables with the top five contributing variables being canopy and sagebrush cover, terrain roughness and wetness, and Gross Primary Productivity. The summarized landscape surface (landsum) had a lower fit (∆AIC = 81.80), but was better than distance alone and included the sum of resistances for higher percentages of tilled agricultural fields, canopy cover, and human disturbance, as well as increased terrain roughness and steepness and a higher annual drought index.

**Figure B.2.** The best establish landscape resistance surface for MZII was the breeding habitat model (BH_50_20) where the predicted breeding habitat probabilities below 0.50 were designated as non-habitat and set to a resistance of 20. The breeding habitat model was comprised of a variety of landscape variables with the top five contributing variables being sagebrush and canopy cover, annual drought index, degree days above 5 ˚C and mean annual precipitation. The summarized landscape surface had a lower fit (∆AIC = 57.65), but was better than distance alone and included the sum of resistances for lower percentages of sagebrush cover, and higher percentages of canopy and tilled agricultural fields, as well as, increased terrain roughness and steepness and a higher number of degree days above 5 ˚C

**Figure B.3.** The best establish landscape resistance surface for MZIII was the breeding population index (BPI_30_010) where values in the lowest 30% were designated as non-habitat and set to a resistance of 10. The population index was comprised of a breeding habitat model (Top 5 variables: sagebrush cover, degree days > 5 ˚C, elevation, Annual Drought Index and canopy cover) and population abundance estimated through lek counts. The model had a slightly lower fit than distance alone (∆AIC = 0.56). The summarized landscape surface (landsum) had a comparatively lower fit than distance (∆AIC = 2.07), and only included terrain roughness. Overall, these results suggest that further analysis is required to develop a better landscape model.

**Figure B.4.** The best establish landscape resistance surface for MZIV was the breeding population index (BPI_50_010) where values in the lowest 50% were designated as non-habitat and set to a resistance of 10. The population index was comprised of a breeding habitat model (Top 5 variables: all sagebrush cover, Annual Drought Index, low sagebrush cover, mean annual precipitation, and degree days > 5 ˚C) and population abundance estimated through lek counts.

The summarized landscape surface (landsum) had a lower fit (∆AIC = 80.78), but was better than distance alone and included the sum of resistances for low sagebrush cover, high tilled agriculture, canopy cover, and human disturbance, as well as rough and steep terrain.

**Figure B.5.** The best establish landscape resistance surface for MZV was the summarized landscape surface (landsum) and included the sum of resistances for low sagebrush cover, high tilled agriculture, canopy cover, and human disturbance, as well as, rough and steep terrain. The breeding population index (BPI_50_010) where values in the lowest 50% were designated as non-habitat and set to a resistance of 10 had a slightly worse fit (∆AIC = 2.61), but was better than distance alone. The population index was comprised of a breeding habitat model (Top 5 variables: elevation, degree days > 5 ˚C, grassland herbaceous cover, Annual Drought Index, and all sagebrush cover) and population abundance estimated through lek counts.
